# Effects of outcome uncertainty related to gain and loss, performance feedback, and individual differences during a cognitive control task

**DOI:** 10.3389/fpsyg.2025.1469701

**Published:** 2025-07-18

**Authors:** Nicolo Biagi, Phoenix Byrne, Jayne Morriss

**Affiliations:** ^1^Henley Business School, Business Informatics Systems and Accounting, Informatics Research Centre, University of Reading, Reading, United Kingdom; ^2^Centre for Integrative Neuroscience and Neurodynamics, School of Psychology and Clinical Language Sciences, University of Reading, Reading, United Kingdom; ^3^School of Psychology, Faculty of Environmental and Life Sciences, University of Southampton, Southampton, United Kingdom

**Keywords:** outcome uncertainty, performance feedback, gain, loss, individual differences, cognitive control, task performance, self-report

## Abstract

**Introduction:**

Outcome uncertainty is known to engage motivational and emotional phenomena. However, there remain questions as to how outcome uncertainty related to gain and loss and information availability via performance feedback interact to impact motivational and emotional phenomena: (1) generally, and (2) in relation to individual differences in intolerance of uncertainty, and current symptoms related to anxiety and depression.

**Methods:**

To address these gaps in the literature, we manipulated the level of outcome uncertainty (gain, loss, none) and performance feedback (present, absent) during an online cognitive control task (*n* = 69), to examine how these factors impact different read-outs: subjective emotional responses (valence, arousal), task accuracy, reaction times, and fixation count. Self-reported intolerance of uncertainty and symptoms of general distress, anxious arousal, and anhedonic depression were also collected.

**Results:**

Outcome uncertainty related to loss and gain, compared to no outcome, was associated with higher arousal and higher task accuracy. Uncertainty about task performance through the absence of performance feedback lowered arousal, dampened positive affect, and led to demotivation (i.e. lowered task accuracy and fixation count). Individual differences in intolerance of uncertainty and symptoms of anxiety and depression were specifically associated with different self-reported experiences of emotion (i.e. valence) and motivational engagement (i.e. fixation count).

**Discussion:**

These findings suggest that outcome uncertainty and performance feedback, as well as intolerance of uncertainty, and anxious/depressive traits differently impact motivation and emotion.

## Introduction

Motivation and emotion are critical for health and wellbeing (Kringelbach and Phillips, 2014). Motivation is generally divided into two systems, which are thought to promote survival (Dickinson and Dearing, 1979). An aversive system supporting avoidance of punishment or negative stimuli (e.g., pain, social rejection, monetary loss) and an appetitive system supporting approach toward potentially rewarding or positive stimuli (e.g., food, sex, social intimacy, monetary gain) (Lang and Bradley, 2010). Internal or external motivationally relevant events evoke emotional reactions, consisting of changes in subjective experience, behavior, and physiological responding (Frijda, 1986; Lang and Bradley, 2010). Despite these advances, there remain gaps in our knowledge as to how contextual dimensions (for commentary see, Cowen and Keltner, 2017) such as uncertainty modulate motivational and emotional phenomena.

Uncertainty refers to “when something is unknown, or when there is a lack of information concerning the probability of future events and their possible outcomes” (e.g., waiting to hear the outcome of a job interview, academic exam, or marriage proposal) (Morriss et al., 2022). The dominant view is that uncertainty is more likely to engage the aversive system associated with negative emotional states (e.g., fear, anxiety) (Brosschot et al., 2016; Gray, 1990; Hirsh et al., 2012; Peters et al., 2017). Indeed, the majority of empirical research on the topic has demonstrated how uncertain negative outcomes, compared to neutral outcomes, result in greater subjective experience of distress, heightened physiological arousal (e.g., skin conductance, pupil dilation, and facial electromyography) and neural activity associated with salience (for review see Grupe and Nitschke, 2013). In a recent survey-based study, uncertainty in general was found to evoke more negative emotional states than positive emotional states (Morriss et al., 2022). Notably, some research suggests that uncertainty does engage the appetitive system as well. For instance, uncertain positive outcomes, compared to neutral outcomes, result in greater physiological arousal (e.g., skin conductance) and salience-related neural activation (Foti and Hajcak, 2012; Lin et al., 2015; Morriss et al., 2019, 2021). Furthermore, a survey-based study revealed that uncertainty, in combination with positive outcomes, was found to elicit both negative and positive emotional states equally (Morriss et al., 2022).

In the literature so far, efforts have generally focused on separately examining uncertainty in relation to negative vs. neutral outcomes, or positive vs. neutral outcomes, under passive viewing (for review, see Morriss et al., 2019). Furthermore, prior research examining uncertainty with mixed valence outcomes under task demand have tended to use uncertain outcomes that are symmetrical in terms of valence (e.g., gain and loss), without a control condition (e.g., no outcome) (Hein et al., 2021; Hübner and Schlösser, 2010). Based on this, it is difficult to compare whether outcome uncertainty related to negative and positive events differentially modulates motivational and emotional phenomena. For instance, does outcome uncertainty related to gain and loss engage motivation equally and heighten negative and/or positive emotional experiences differently?

It is well established that both animals and humans are driven to identify and seek out information in their environment, in order to reduce or resolve uncertainty (Peters et al., 2017). Cognitive control is the coordination of cognitive processes to attain goals (Miller, 2000) and it is essential for selecting and filtering information to reduce uncertainty (Del Popolo Cristaldi et al., 2023; Fan, 2014; Wu et al., 2020, 2021). Factors such as the availability of information likely contribute to uncertainty in relation to motivational and emotional phenomena. The availability of information via performance feedback (e.g., correct, incorrect) has been extensively studied using cognitive control tasks (for review see Mushtaq et al., 2011). An earlier study demonstrated that the absence (e.g., increases uncertainty about performance), compared to the presence of performance feedback resulted in poorer task accuracy and slower reaction times (Moret-Tatay et al., 2016). Although, to date only a few studies using cognitive control tasks have directly manipulated uncertainty (Jackson et al., 2015; Sandre and Weinberg, 2019; Speed et al., 2017). Two studies reported that task irrelevant uncertainty (e.g., unpredictable tones) increased accuracy and neural markers associated with error monitoring (Jackson et al., 2015; Speed et al., 2017). Another study that included ambiguous trials without a correct or incorrect answer did not find any differences in accuracy but observed greater changes in neural markers associated with error monitoring (Sandre and Weinberg, 2019). Alongside these findings, there is also evidence that under high levels of uncertainty, there is a greater desire for performance related feedback (Anseel and Lievens, 2007). In sum, these findings suggest that during cognitive control tasks uncertainty increases task performance and error monitoring, and that uncertainty may be reduced via performance feedback. Notably, it has been largely unexplored as to how interactions between task relevant uncertainty related to positive and negative events and information availability via performance feedback impact motivational and emotional phenomena. For example, does the outcome uncertainty related to gain and loss and the presence or absence of performance feedback interact to modulate emotional and motivational phenomena differently?

Previous research has demonstrated that sensitivity to uncertain valenced (e.g., reward, punishment) outcomes is associated with individual differences in intolerance of uncertainty (e.g., tendency to find uncertainty aversive, for review see Morriss et al., 2021, 2023a,b; Sandhu et al., 2023; Tanovic et al., 2018) and anxiety/depressive symptomology (Pulcu and Browning, 2019; Brown et al., 2023). More specifically for example, under uncertain threat, even when mild, higher intolerance of uncertainty is associated with greater attentional deployment (Del Popolo Cristaldi et al., 2021), arousal (Morriss et al., 2016), and information seeking behavior (Bartoszek et al., 2022; Wake et al., 2022). However, at this current time, there has been a lack of research on how uncertainty related to performance feedback (e.g., the absence of this information), is associated with individual differences in intolerance of uncertainty and anxiety/depressive symptomology. Relatedly, very little research has explored whether interactions between outcome uncertainty related to positive and negative events and information availability via performance feedback are influenced by individual differences in intolerance of uncertainty and current experiences of anxiety and depressive symptoms.

The present experiment aimed to address these gaps in the literature by examining how outcome uncertainty related to gain and loss, performance feedback, and individual differences (intolerance of uncertainty/anxious and depressive symptoms) during an online cognitive control task (modified Eriksen Flanker) impact different measurements of motivational and emotional phenomena: self-reported emotional states (valence, arousal), task performance (accuracy, reaction time), and fixation count. Outcome uncertainty related to gain and loss was manipulated via instructions before each experimental block that stated whether task performance would result in a potential monetary gain, a potential monetary loss, or that there was no possibility of monetary gain or loss. Information availability via performance feedback was manipulated such that half of the experimental blocks included the presence of feedback (i.e., “correct”, “Incorrect”) and half of the experimental blocks included the absence of feedback (i.e., “?”).

Hypotheses:

1. Outcome uncertainty related to gain or loss, vs. no outcome, will lead to higher affect and motivation (Grupe and Nitschke, 2013; Morriss et al., 2019) e.g., evoke greater subjective ratings of arousal and positive/negative valence, improve task performance (higher accuracy, faster reaction time), and elicit higher fixation count to the flanker and feedback.
2. The lack of possibility to reduce uncertainty related to the absence of performance feedback, compared to the presence of performance feedback, will lead to higher negative affect and decreased motivation (Morriss et al., 2022) e.g., evoke larger subjective ratings of negative affect, impair task performance (lower accuracy, slow reaction times; see Moret-Tatay et al., 2016) and lower fixation count to the flanker and feedback.
3. Outcome uncertainty related to gain and loss and performance feedback may interact to impact task performance, subjective ratings of emotional experience, and fixation count. However, given the lack of prior research on the topic, the direction and strength of such an interaction is currently unclear.
4. Outcome uncertainty related to gain and loss, relative to no outcome, may lmodulate arousal, valence, and motivation based on individual differences in intolerance of uncertainty (for review see Tanovic et al., 2018). The absence of performance feedback in combination with outcome uncertainty related to gain and loss may exacerbate such effects (Morriss et al., 2023a,b).
5. Interactions between the type of outcome uncertainty and performance feedback may be associated with current symptoms of anxious arousal/anhedonic depression. Anxious arousal may be related to increased negative affect and motivation during the task, particularly under conditions with potential loss (Mogg and Bradley, 1998). Anhedonic depression may be related to decreased positive affect and motivation during the task, particularly under conditions with potential gain (Treadway and Zald, 2011).

## Materials and methods

### Participants

Data was obtained from a total of 79 participants, 5 were excluded due to absent responding and a further 5 for having a performance rate below 60%. Consequently, the final sample consisted of 69 participants (36 Female, 32 Male, 1 Not specified) aged between 18 and 41 (*M* = 25.66 years, *SD* = 5.13). Further demographic information from 68 participants can be found in Table 1. Participants were eligible to take part provided they were within the specified age range, per above, and had access to a working webcam. Participant recruitment consisted of the dissemination of posters, published across the University of Reading and various social medial platforms (e.g., Facebook, Twitter, Instagram). Information regarding past and/or present psychopathology was not collected, nor controlled. Although involvement was voluntary, participants were given a £10 Amazon voucher as a reimbursement for their time. Note, participants were informed that they would receive a £5 Amazon voucher, plus the opportunity to win more, contingent on task performance. However, irrespective of performance, all participants received a £10 voucher. The study procedure was approved by the University of Reading Research Ethics Committee.

**Table 1:** Overview of additional demographic information.

	***n***	**%**
**Ethnicity**		
White	39	57.35 %
Black	12	17.65 %
Multi-ethnic	9	13.24 %
Asian	4	5.88 %
Middle Eastern	2	2.94 %
Other	1	1.47 %
Unspecified	1	1.47 %
**English level**		
First language	58	85.29 %
Second language	8	11.76 %
Unspecified	2	2.94 %
**Nationality**		
European	54	79.41 %
North American	6	8.82 %
Asian	3	4.41 %
South American	1	1.47 %
Multi-nationality	1	1.47 %
Unspecified	3	4.41 %
**Sexual orientation**		
Heterosexual	53	80.88 %
LGBTQ+	12	17.65 %
Unspecified	3	4.41 %

Sample size was informed by a power analysis for a repeated measures ANOVA conducted in G^*^Power 3 (Faul et al., 2007), to examine the main/interaction effects of uncertainty and valence: *f* = 0.15, α error probability = 0.05, Power (1 – β error probability) = 0.8, number of groups = 1, number of measurements = 6 [(2: present, absent) × 3 (gain, loss, none)], correlation among repeated measures = 0.3. This analysis resulted in a sample size suggestion of 68 participants. A small-medium effect size was entered due to the lack of research examining the impact of uncertainty × valence interactions during a cognitive control task. Notably, similar sample sizes (e.g., 60–70) have been commonly used in studies to explore the impact of individual differences in intolerance of uncertainty and anxious temperament on behavioral and psychophysiological measures (Klingelhöfer-Jens et al., 2022; Rodriguez-Sobstel et al., 2023).

### Procedure

All experimental phases were completed using a PC or laptop online. Informed consent was obtained in the first instance, prior to the start of the task. Participants then received task instructions, see Flanker Task section for more details. Subsequently, participants were prompted to set-up their webcam and position themselves appropriately to enable eye-tracking throughout (for more detail, refer to Eyetracking section). Once optimal, participants were exposed to the eye-tracking calibration and validation, consisting of two phases. The first phase instructed participants to move their eyes to follow the location of a single black dot presented on screen, whilst clicking the dot simultaneously. The second phase resembled the first, however, participants were required to follow the dot only, without clicking. Successful calibration led participants into the main Flanker Task.

An experimenter was present during task set-u*p* to assist and answer any questions, however, exited prior to starting the Flanker Task. Once the task was completed, participants were given demographic questions (i.e., age, ethnicity, gender etc.) and questionnaires (see Questionnaires) to complete. The task, including set-up, took ~30 min.

### Online flanker task

A modified version of the Eriksen flanker task (Eriksen and Schultz, 1979) was used. This task was built using JsPych (de Leeuw, 2015) and consisted of 240 trials in total, divided into 6 blocks of 40 trials. Each block presented the opportunity to either gain or lose real voucher money contingent on task performance, with a further block condition offering no gain or loss. Each of these blocks were delivered twice, equating to 80 trials per valence. Block order was counterbalanced, resulting in 8 block combinations. Each combination was allocated to a group, to which participants were sequentially assigned. Participants were notified if they could win, lose, or win nor lose at the start of each block.

During the task, participants were presented with a central fixation cross for 500 ms, followed by five horizontal arrows, displayed for 800 ms until a response was provided. Participants were instructed to attend to the central arrow, whilst ignoring the four adjacent flankers. When exposed to the arrow array, participants were asked to indicate the direction of the central arrow, by pressing the corresponding arrow key on their keyboard i.e., press the left key for a left facing central arrow or press the right key for a right facing arrow. Each trial was either congruent or incongruent, split 50/50, thus, 120 trials each. For congruent, the flanking arrows all pointed in the same direction as the target arrow (i.e., < < < < < ). For incongruent, the flanking arrows all pointed in the opposite direction (i.e., < < > < < ). Within each array, arrows were equally sized and distanced. Once a response had been selected, participants received performance feedback. This feedback was either present (i.e., given legitimate feedback such as “correct” or “incorrect”) or absent (i.e., they were given a “?”). These were split equally across the task (120 trials each). Trial order was pseudorandomized, with the constraint that there be no more than three of the same trial type presented consecutively.

At the end of each block, participants were asked to rate valence (1 = negative, 9 = positive), arousal (1 = Calm, 9 = Excited) and felt emotions (excited, enthusiastic, happy, joyful, sad, upset, angry, frustrated, fearful and anxious). For felt emotions, participants were instructed to rate the intensity of each emotion category from 0 to 6 (i.e., 0 = not sad at all, 6 = being very sad). The felt emotions are not reported in this manuscript as they were not central to the hypotheses and were recorded for exploratory purposes.

### Eye-tracking

Eye-tracking data were collected throughout the task using the WebGazer library (Papoutsaki et al., 2016), contained within jsPsych. Utilizing a webcam, Webgazer implements vision techniques to detect and analyze specific visual attributes of a participant's eyes to forecast the individual's point of gaze. Before the start of the task, a 5-point calibration and a 5-point validation was carried out to maximize the quality of the data (see Procedure for more details).

### Questionnaires

The *Intolerance of Uncertainty Scale*−12 item (IUS, Freeston et al., 1994; Carleton et al., 2007) consists of 12-items which are rated on a 5 point scale (1 “not at all typical of me” to 5 “very typical of me”). Participants were instructed to score each item based on how characteristic it is for them. The IUS is scored by creating a total score of all the items. IUS achieved an internal consistency of α = 0.89.

The *Mini Mood and Anxiety Symptom Questionnaire* (Mini-MASQ, Clark and Watson, 1991), consists of 26 items, which are rated on a 5 point-scale (1 = “Not at all” to 5 = “Extremely”). The Mini-MASQ includes three subscales: (1) General Distress (GD, 8 items), (2) Anxious Arousal (AA, 10 items), and (3) Anhedonic Depression (AD, 8 items). Participants were instructed to score each item based on how much they “have felt or experienced things this way during the past week, including today”. The Mini-MASQ is scored by creating a total score of all the items for each subscale. The Mini-MASQ, GD, AA and AD subscales yielded the following internal consistency: α = 0.9, α = 0.78, and α = 0.86, respectively.

### Data reduction

#### Reaction time

An analysis of reaction time (RT) during the flanker task was conducted. Originally, the dataset consisted of 18,960 trials, however, only correct trials with an RT between 250 and 800 ms were retained. This reduced the number of trials included in the analysis to 15,644 [82.5%]. Subsequently, a grand average for each participant across all combinations of outcome uncertainty (loss, gain, none), performance feedback (present, absent), and trial type (congruent and incongruent) was calculated.

#### Accuracy

An analysis of accuracy was also conducted. The original dataset comprised of 18,960 trials, with 16,267 [85.8%] kept for further analysis. The percentage of correct trials for each participant across all combinations of outcome uncertainty (loss, gain, none), performance feedback (present, absent), and congruency (congruent and incongruent) was calculated.

#### Fixation count

Depending on the device used by the participant, the frequency of sampling varied. For this reason, the number of times the eye-tracker samples were recorded inside an “area of interest” was used. The eyetracker data consisted of × and *Y* coordinates (in screen pixels) and a t variable to indicate the timestamp. To ensure the data from different devices was comparable, the eye-tracker data were converted from screen pixels to percentage of screen resolution.

Initially, samples with a × or *Y* percentage smaller than 0 or bigger than 100 (i.e., non-valid eye data samples) were removed, reducing the dataset to 886,295 samples. For all samples, the “area of interest” count was calculated, described above. The “area of interest” included the full string for the flanker or the full string for the feedback, and was calculated as a × and Y percentage bigger than 40 and smaller than 60. Subsequently, the number of samples (i.e., Fixation Count) within the “area of interest” for each participant across all combinations of outcome uncertainty (loss, gain, none), performance feedback (present, absent), and trial type (congruent and incongruent) was calculated, reducing the dataset to 270,906 samples [30.5%]. This calculation was done for the flanker and feedback phases. The number of samples for the flanker presentation was 174,768, of which 54,242 [30%] were valid samples. The number of samples for the feedback display was 348,707, of which 105,182 [31%] were valid samples. To clarify, 30% of data points fell within the area of interest, the other remaining 70% were not of interest (rest of the computer screen).

For 8 participants, Webgazer did not produce usable results, meaning they were excluded from the analysis. This reduced the sample size to 56 participants for fixation count.

### Analysis plan

To explore the effects of Outcome Uncertainty (Gain, Loss, No Outcome) and Performance Feedback (Present, Absent) on affective responding, multilevel models (MLMs) were conducted separately for valence ratings and arousal ratings. Similarly, to assess the effects of Outcome Uncertainty, Performance Feedback and Congruency (Congruent, Incongruent) on task performance, MLMs were run for reaction time, accuracy, and fixation count. All experimental manipulations were within-subjects factors. Demeaned IUS, MASQ-AD, MASQ-GD and MASQ-AA were included in all models to assess individual differences. All models included all possible interactions among the experimental factors and the individual difference measures. Participant ID was modeled as a random intercept to account for within-subject variability. Restricted maximum likelihood estimation was disabled to allow for model comparison.

Estimated marginal means (EMMs) were used for significant main and interaction effects between factors, where appropriate. To further visualize any significant interactions between a covariate and factor, simples slopes analysis were performed and subsequently, entered into pairwise comparisons. When applicable, multiple comparisons were Tukey's-adjusted.

## Results

### Main effects

#### Valence ratings

**Figure 1:**
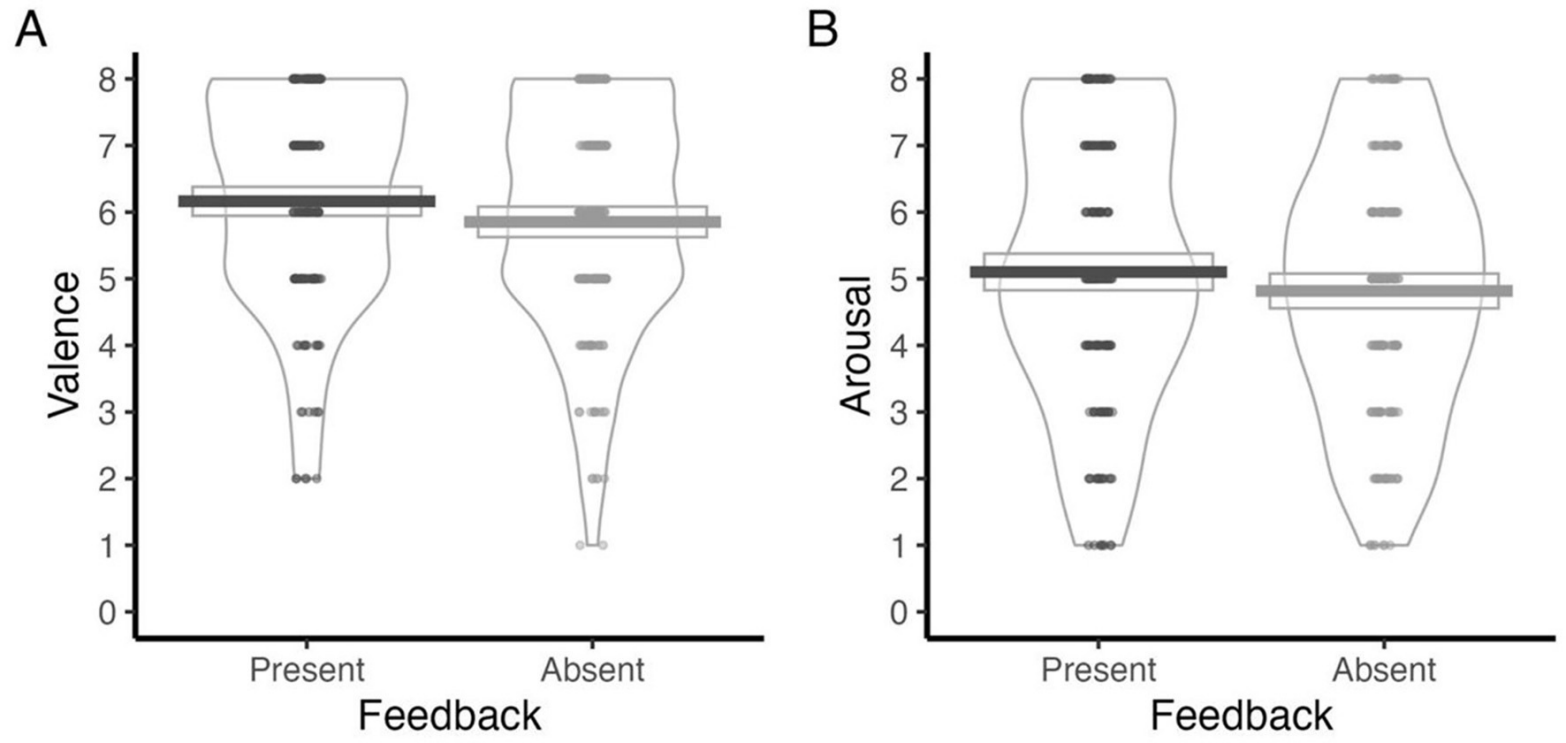
Valence **(A)** and Arousal **(B)** ratings for feedback-present and feedback-absent trials. Note: Valence and Arousal ratings were indicated on a 1–9 scale. For Valence, 1 = negative and 9 = positive. For Arousal, 1 = calm and 9 = excited.

Analyses revealed a significant main effect of Performance Feedback on valence ratings (Table 2). Specifically, participants reported more positive valence ratings in the presence of feedback (*M* = 6.82, SD = 1.84) compared to when it was absent (*M* = 6.4, SD = 1.94) (Figure 1A).

**Table 2:** MLM analyses for valence and arousal ratings.

**Model**	**Effect**	**d*f***	***F***	***p***
**Valence**	Performance Feedback	1, 345	14.69	< 0.001^*^
	Outcome Uncertainty	2, 345	2.68	0.07
	IUS	1, 69	0	0.98
	MASQ-AA	1, 69	2.13	0.15
	MASQ-GDD	1, 69	0.77	0.38
	MASQ-AD	1, 69	1.41	0.001^*^
	Performance Feedback × Outcome Uncertainty	2, 345	0.86	0.42
	Performance Feedback × IUS	1, 345	0.23	0.63
	Performance Feedback × MASQ-AA	1, 345	1.52	0.22
	Performance Feedback × MASQ-GDD	1, 345	0.17	0.68
	Performance Feedback × MASQ-AD	1, 345	0.1	0.76
	Outcome Uncertainty × IUS	2, 345	3.77	0.02^*^
	Outcome Uncertainty × MASQ-AA	2, 345	2.11	0.12
	Outcome Uncertainty × MASQ-GDD	2, 345	1.71	0.18
	Outcome Uncertainty × MASQ-AD	2, 345	1.64	0.19
	Performance Feedback × Outcome Uncertainty × IUS	2, 345	3.16	0.04^*^
	Performance Feedback × Outcome Uncertainty × MASQ-AA	2, 345	1.46	0.23
	Performance Feedback × Outcome Uncertainty × MASQ-GDD	2, 345	1.59	0.21
	Performance Feedback × Outcome Uncertainty × MASQ-AD	2, 345	1.42	0.24
**Arousal**	Performance Feedback	1, 345	8.94	002^*^
	Outcome Uncertainty	2, 345	8.07	< 0.001^*^
	IUS	1, 69	0.08	0.78
	MASQ-AA	1, 69	2.12	0.15
	MASQ-GDD	1, 69	0	0.98
	MASQ-AD	1, 69	0.77	0.38
	Performance Feedback × Outcome Uncertainty	2, 345	0.35	0.70
	Performance Feedback × IUS	1, 345	1.4	0.24
	Performance Feedback × MASQ-AA	1, 345	2.01	0.16
	Performance Feedback × MASQ-GDD	1, 345	0	0.97
	Performance Feedback × MASQ-AD	1, 345	0.05	0.83
	Outcome Uncertainty × IUS	2, 345	1.09	0.34
	Outcome Uncertainty × MASQ-AA	2, 345	1.66	0.19
	Outcome Uncertainty × MASQ-GDD	2, 345	0.62	0.54
	Outcome Uncertainty × MASQ-AD	2, 345	1.05	0.35
	Performance Feedback × Outcome Uncertainty × IUS	2, 345	0.84	0.43
	Performance Feedback × Outcome Uncertainty × MASQ-AA	2, 345	0.17	0.85
	Performance Feedback × Outcome Uncertainty × MASQ-GDD	2, 345	0.78	0.46
	Performance Feedback × Outcome Uncertainty × MASQ-AD	2, 345	1.25	0.28

Significant effects are denoted with an asterisk.

#### Arousal ratings

Consistent with the valence findings, a significant main effect of Performance Feedback was also observed for arousal ratings (Table 2), with elevated arousal reported when feedback was provided (*M* = 5.69, SD = 2.33), relative to when it was not (*M* = 5.34, SD = 2.26) (Figure 1B).

**Figure 2:**
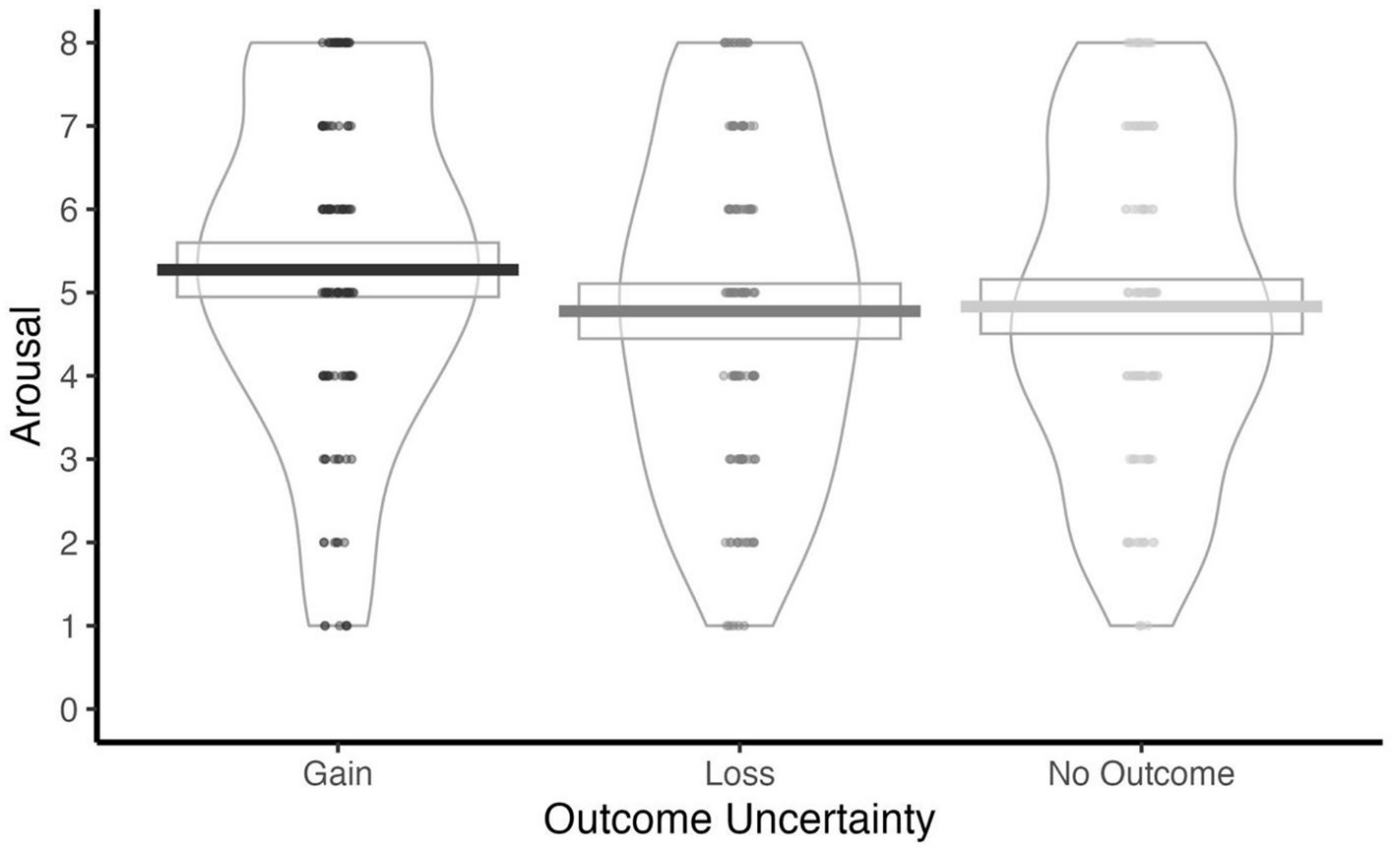
Arousal ratings for Gain, Loss, and No Outcome blocks. Note: Arousal was reported from 1 = calm and 9 = excited.

A significant main effect of Outcome Uncertainty was also identified (Table 2). *Post-hoc* analysis of estimated marginal means revealed that arousal was lowest during loss blocks (*M* = 5.30, SE = 0.25, 95% CI [4.79, 5.80]), modestly higher in the No Outcome context (*M* = 5.41, SE = 0.25, 95% CI [4.90, 5.91]), and highest during Gain blocks (*M* = 5.84, SE = 0.25, 95% CI [5.34, 6.34]). Pairwise comparisons indicated significantly reduced arousal in the loss condition relative to the Gain condition, *b* = −0.543, SE = 0.148, *t*_(372)_ = −3.662, *p* < 0.001. A similar reduction was observed in the No Outcome vs. Gain comparison, *b* = −0.435, SE = 0.148, *t*_(372)_ = −2.929, *p* = 0.01. No significant difference emerged between the Loss and No Outcome conditions, *b* = −0.109, SE = 0.148, *t*_(372)_ = −0.732, *p* = 0.744 (see Figure 2).

#### Flanker fixation

**Figure 3:**
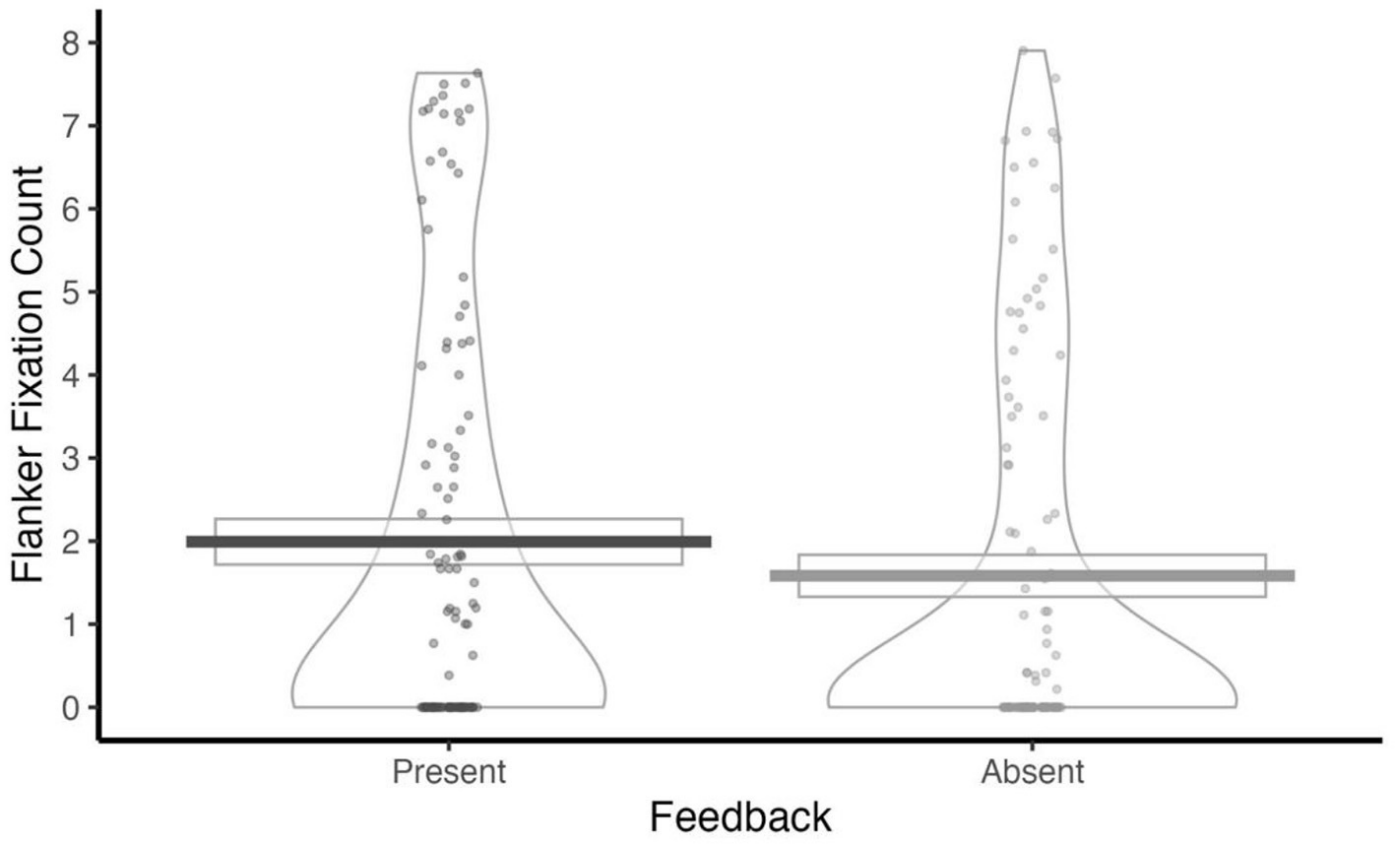
Flanker Fixation count at each level of Performance Feedback (Present, Absent).

As shown in Table 3, a significant main effect of Performance Feedback on flanker fixation was observed. Participants made more flanker fixations when feedback was present (*M* = 32.97, SD = 32.26) than when it was absent (*M* = 30.47, SD = 31.40) (Figure 3).

**Table 3:** MLM analyses for flanker fixation and feedback fixation.

**Model**	**Effect**	**d*f***	***F***	***p***
**Flanker Fixation**	Performance Feedback	1, 603.01	6.11	0.01^*^
	Outcome Uncertainty	2, 603.01	2.49	0.08
	Congruency	1, 603.01	0.02	0.88
	IUS	1, 55	0.03	0.87
	MASQ-AA	1, 55	8.83	0.004^*^
	MASQ-GDD	1, 55	2.22	0.14
	MASQ-AD	1, 55	1.54	0.22
	Performance Feedback × Outcome Uncertainty	2, 603.01	2.42	0.09
	Performance Feedback × Congruency	1, 603.01	0	0.98
	Outcome Uncertainty × Congruency	2, 603.01	0.17	0.84
	Performance Feedback × IUS	1, 603.01	0.05	0.83
	Performance Feedback × MASQ-AA	1, 603.01	2.69	0.1
	Performance Feedback × MASQ-GDD	1, 603.01	0.14	0.71
	Performance Feedback × MASQ-AD	1, 603.01	0.67	0.41
	Outcome Uncertainty × IUS	2, 603.01	1.54	0.21
	Outcome Uncertainty × MASQ-AA	2, 603.01	3.65	0.03^*^
	Outcome Uncertainty × MASQ-GDD	2, 603.01	8.63	< 0.001^*^
	Outcome Uncertainty × MASQ-AD	2, 603.01	5.78	0.003^*^
	Congruency × IUS	1, 603.01	0.51	0.47
	Congruency × MASQ-AA	1, 603.01	0.35	0.55
	Congruency × MASQ-GDD	1, 603.01	0.23	0.63
	Congruency × MASQ-AD	1, 603.01	0.3	0.58
	Performance Feedback × Outcome Uncertainty × Congruency	2, 603.01	0.39	0.68
	Performance Feedback × Outcome Uncertainty × IUS	2, 603.01	1.71	0.18
	Performance Feedback × Outcome Uncertainty × MASQ-AA	2, 603.01	5.31	0.005^*^
	Performance Feedback × Outcome Uncertainty × MASQ-GDD	2, 603.01	1.08	0.34
	Performance Feedback × Outcome Uncertainty × MASQ-AD	2, 603.01	3.05	0.05^*^
	Performance Feedback × Congruency × IUS	1, 603.01	0.01	0.92
	Performance Feedback × Congruency × MASQ-AA	1, 603.01	0.1	0.75
	Performance Feedback × Congruency × MASQ-GDD	1, 603.01	0.08	0.78
	Performance Feedback × Congruency × MASQ-AD	1, 603.01	0.14	0.71
	Outcome Uncertainty × Congruency × IUS	2, 603.01	0.15	0.86
	Outcome Uncertainty × Congruency × MASQ-AA	2, 603.01	0	0.99
	Outcome Uncertainty × Congruency × MASQ-GDD	2, 603.01	0.05	0.95
	Outcome Uncertainty × Congruency × MASQ-AD	2, 603.01	0.02	0.98
	Performance Feedback × Outcome Uncertainty × Congruency × IUS	2, 603.01	0.04	0.96
	Performance Feedback × Outcome Uncertainty × Congruency × MASQ-AA	2, 603.01	0.02	0.98
	Performance Feedback × Outcome Uncertainty × Congruency × MASQ-GDD	2, 603.01	0.09	0.91
	Performance Feedback × Outcome Uncertainty × Congruency × MASQ-AD	2, 603.01	0.09	0.91
**Feedback fixation**	Performance Feedback	1, 605	2.62	0.11
	Outcome Uncertainty	2, 605	2.23	0.11
	Congruency	1, 605	0.24	0.62
	IUS	1, 55	0.02	0.89
	MASQ-AA	1, 55	8.22	0.005^*^
	MASQ-GDD	1, 55	2.87	0.09
	MASQ-AD	1, 55	2.22	0.14
	Performance Feedback × Outcome Uncertainty	2, 605	2.39	0.09
	Performance Feedback × Congruency	1, 605	0.14	0.71
	Outcome Uncertainty × Congruency	2, 605	0.07	0.93
	Performance Feedback × IUS	1, 605	0.36	0.55
	Performance Feedback × MASQ-AA	1, 605	2.92	0.08
	Performance Feedback × MASQ-GDD	1, 605	0.67	0.41
	Performance Feedback × MASQ-AD	1, 605	1.42	0.23
	Outcome Uncertainty × IUS	2, 605	1.71	0.18
	Outcome Uncertainty × MASQ-AA	2, 605	2.99	0.05
	Outcome Uncertainty × MASQ-GDD	2, 605	3.64	0.03^*^
	Outcome Uncertainty × MASQ-AD	2, 605	3.61	0.03^*^
	Congruency × IUS	1, 605	0.01	0.9
	Congruency × MASQ-AA	1, 605	1.17	0.28
	Congruency × MASQ-GDD	1, 605	0.21	0.65
	Congruency × MASQ-AD	1, 605	0.04	0.83
	Performance Feedback × Outcome Uncertainty × Congruency	2, 605	0.26	0.77
	Performance Feedback × Outcome Uncertainty × IUS	2, 605	2.04	0.13
	Performance Feedback × Outcome Uncertainty × MASQ-AA	2, 605	3.95	0.019^*^
	Performance Feedback × Outcome Uncertainty × MASQ-GDD	2, 605	0.93	0.39
	Performance Feedback × Outcome Uncertainty × MASQ-AD	2, 605	1.18	0.3
	Performance Feedback × Congruency × IUS	1, 605	0.02	0.89
	Performance Feedback × Congruency × MASQ-AA	1, 605	0	0.99
	Performance Feedback × Congruency × MASQ-GDD	1, 605	0.38	0.54
	Performance Feedback × Congruency × MASQ-AD	1, 605	0.13	0.72
	Outcome Uncertainty × Congruency × IUS	2, 605	0.02	0.97
	Outcome Uncertainty × Congruency × MASQ-AA	2, 605	0.17	0.84
	Outcome Uncertainty × Congruency × MASQ-GDD	2, 605	0.12	0.88
	Outcome Uncertainty × Congruency × MASQ-AD	2, 605	0.14	0.87
	Performance Feedback × Outcome Uncertainty × Congruency × IUS	2, 605	0	0.99
	Performance Feedback × Outcome Uncertainty × Congruency × MASQ-AA	2, 605	0.08	0.93
	Performance Feedback × Outcome Uncertainty × Congruency × MASQ-GDD	2, 605	0.23	0.79
	Performance Feedback × Outcome Uncertainty × Congruency × MASQ-AD	2, 605	0.47	0.62

Significant effects are denoted with an asterisk.

#### Feedback fixation

No significant main or interaction effects were observed for Performance Feedback, Outcome Uncertainty and Congruency (Table 3).

#### Response time

**Figure 4:**
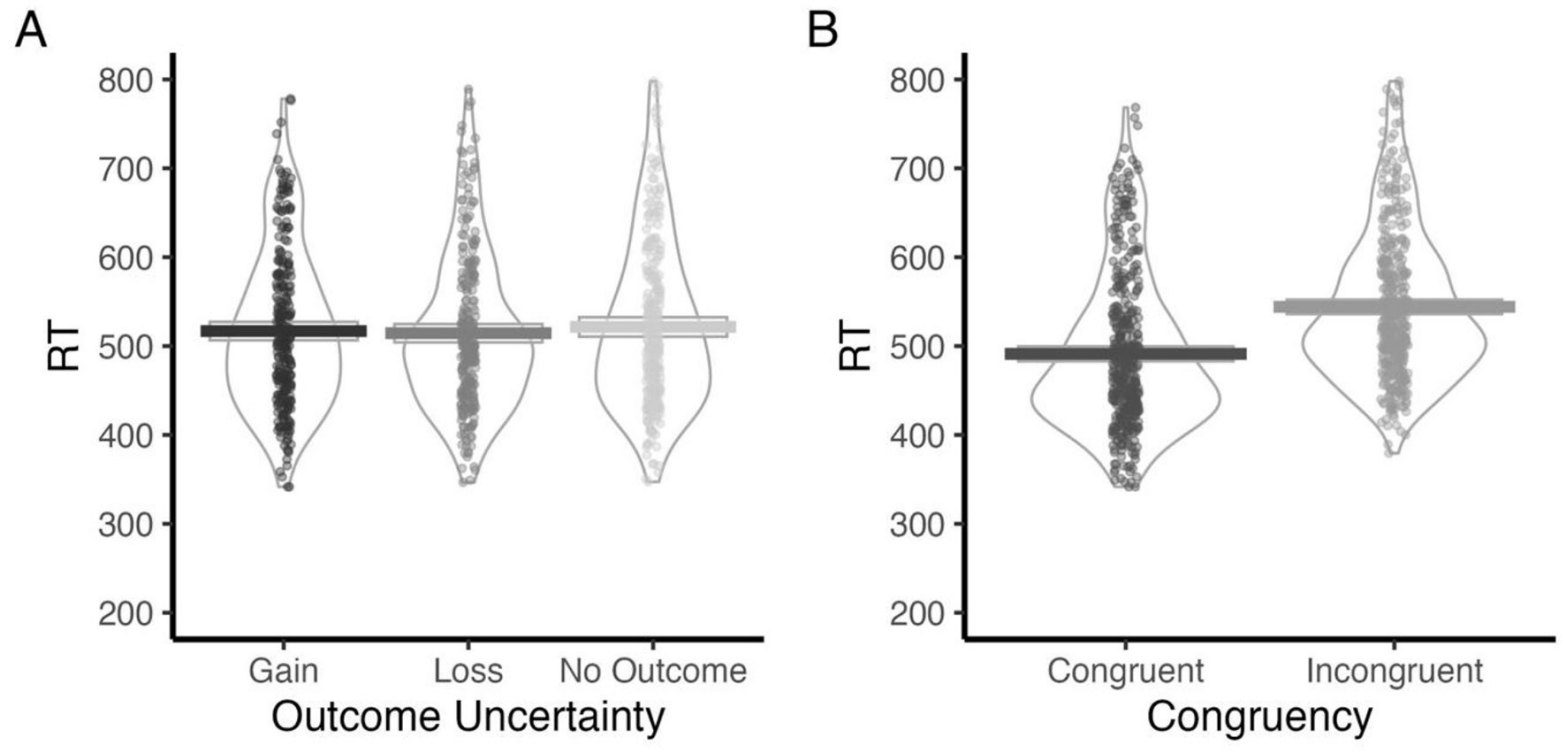
RT for levels of **(A)** Outcome Uncertainty (Gain, Loss, No Outcome), and **(B)** Congruency (Congruent, Incongruent).

Analysis of response times revealed a significant main effect of Outcome Uncertainty (Table 4). Subsequent pairwise comparisons showed that participants responded significantly faster in the Loss blocks (*M* = 516 ms, SE = 9.97) compared to No Outcome blocks (*M* = 525 ms, SE = 9.97), Δ = −9.5, SE = 2.82, *t*_(818)_ = −3.375, *p* = 0.002 (Figure 4A). No significant differences were observed between the Loss and Gain (*p* = 0.41), or No Outcome and Gain (*p* = 0.09) conditions.

**Table 4:** MLM analyses for RT and accuracy.

**Model**	**Effect**	**d*f***	***F***	***p***
**RT**	Performance Feedback	1, 759	2.46	0.12
	Outcome Uncertainty	2, 759	6.48	0.001^*^
	Congruency	1, 759	604.64	< 0.001^*^
	IUS	1, 69	5.46	0.02^*^
	MASQ-AA	1, 69	2.12	0.15
	MASQ-GDD	1, 69	0.15	0.69
	MASQ-AD	1, 69	0.03	0.85
	Performance Feedback × Outcome Uncertainty	2, 759	1.06	0.35
	Performance Feedback × Congruency	1, 759	0.17	0.68
	Outcome Uncertainty × Congruency	2, 759	0.53	0.59
	Performance Feedback × IUS	1, 759	7.54	0.006^*^
	Performance Feedback × MASQ-AA	1, 759	0.02	0.89
	Performance Feedback × MASQ-GDD	1, 759	0.72	0.39
	Performance Feedback × MASQ-AD	1, 759	1.23	0.27
	Outcome Uncertainty × IUS	2, 759	1.48	0.23
	Outcome Uncertainty × MASQ-AA	2, 759	0.53	0.59
	Outcome Uncertainty × MASQ-GDD	2, 759	0.02	0.98
	Outcome Uncertainty × MASQ-AD	2, 759	0.64	0.53
	Congruency × IUS	1, 759	0.83	0.36
	Congruency × MASQ-AA	1, 759	5.98	0.01^*^
	Congruency × MASQ-GDD	1, 759	2.12	0.15
	Congruency × MASQ-AD	1, 759	1.29	0.26
	Performance Feedback × Outcome Uncertainty × Congruency	2, 759	1.21	0.29
	Performance Feedback × Outcome Uncertainty × IUS	2, 759	1.67	0.18
	Performance Feedback × Outcome Uncertainty × MASQ-AA	2, 759	0.24	0.79
	Performance Feedback × Outcome Uncertainty × MASQ-GDD	2, 759	4.01	0.02^*^
	Performance Feedback × Outcome Uncertainty × MASQ-AD	2, 759	3.1	0.05^*^
	Performance Feedback × Congruency × IUS	1, 759	0.51	0.48
	Performance Feedback × Congruency × MASQ-AA	1, 759	0.25	0.62
	Performance Feedback × Congruency × MASQ-GDD	1, 759	1.71	0.19
	Performance Feedback × Congruency × MASQ-AD	1, 759	0.18	0.67
	Outcome Uncertainty × Congruency × IUS	2, 759	0.53	0.59
	Outcome Uncertainty × Congruency × MASQ-AA	2, 759	0.01	0.99
	Outcome Uncertainty × Congruency × MASQ-GDD	2, 759	0.06	0.94
	Outcome Uncertainty × Congruency × MASQ-AD	2, 759	0.39	0.68
	Performance Feedback × Outcome Uncertainty × Congruency × IUS	2, 759	0.17	0.84
	Performance Feedback × Outcome Uncertainty × Congruency × MASQ-AA	2, 759	0.14	0.87
	Performance Feedback × Outcome Uncertainty × Congruency × MASQ-GDD	2, 759	0.13	0.88
	Performance Feedback × Outcome Uncertainty × Congruency × MASQ-AD	2, 759	0.06	0.94
**Accuracy**	Performance Feedback	1, 759	8.7	0.003^*^
	Outcome Uncertainty	2, 759	7.68	< 0.001^*^
	Congruency	1, 759	102.95	< 0.001^*^
	IUS	1, 69	1.79	0.19
	MASQ-AA	1, 69	1.71	0.19
	MASQ-GDD	1, 69	0.91	0.34
	MASQ-AD	1, 69	3.3	0.07
	Performance Feedback × Outcome Uncertainty	2, 759	0.2	0.82
	Performance Feedback × Congruency	1, 759	7.83	0.005^*^
	Outcome Uncertainty × Congruency	2, 759	3.9	0.02^*^
	Performance Feedback × IUS	1, 759	2.47	0.12
	Performance Feedback × MASQ-AA	1, 759	1.46	0.23
	Performance Feedback × MASQ-GDD	1, 759	1.49	0.22
	Performance Feedback × MASQ-AD	1, 759	0.74	0.39
	Outcome Uncertainty × IUS	2, 759	1.12	0.33
	Outcome Uncertainty × MASQ-AA	2, 759	0.38	0.68
	Outcome Uncertainty × MASQ-GDD	2, 759	0.2	0.82
	Outcome Uncertainty × MASQ-AD	2, 759	0.45	0.64
	Congruency × IUS	1, 759	5.47	0.02^*^
	Congruency × MASQ-AA	1, 759	6.34	0.01^*^
	Congruency × MASQ-GDD	1, 759	0.33	0.57
	Congruency × MASQ-AD	1, 759	4.05	0.04^*^
	Performance Feedback × Outcome Uncertainty × Congruency	2, 759	0.79	0.45
	Performance Feedback × Outcome Uncertainty × IUS	2, 759	0.54	0.58
	Performance Feedback × Outcome Uncertainty × MASQ-AA	2, 759	0.15	0.86
	Performance Feedback × Outcome Uncertainty × MASQ-GDD	2, 759	0.4	0.67
	Performance Feedback × Outcome Uncertainty × MASQ-AD	2, 759	0.89	0.41
	Performance Feedback × Congruency × IUS	1, 759	2.58	0.11
	Performance Feedback × Congruency × MASQ-AA	1, 759	2.53	0.11
	Performance Feedback × Congruency × MASQ-GDD	1, 759	0.46	0.49
	Performance Feedback × Congruency × MASQ-AD	1, 759	0	0.95
	Outcome Uncertainty × Congruency × IUS	2, 759	1.01	0.37
	Outcome Uncertainty × Congruency × MASQ-AA	2, 759	0.37	0.69
	Outcome Uncertainty × Congruency × MASQ-GDD	2, 759	0.19	0.82
	Outcome Uncertainty × Congruency × MASQ-AD	2, 759	0.1	0.91
	Performance Feedback × Outcome Uncertainty × Congruency × IUS	2, 759	0.6	0.55
	Performance Feedback × Outcome Uncertainty × Congruency × MASQ-AA	2, 759	0.21	0.81
	Performance Feedback × Outcome Uncertainty × Congruency × MASQ-GDD	2, 759	1.05	0.35
	Performance Feedback × Outcome Uncertainty × Congruency × MASQ-AD	2, 759	0.69	0.5

Significant effects are denoted with an asterisk.

Additionally, a significant main effect of congruency was observed (Table 4): participants responded more quickly in congruent trials (*M* = 492.83 ms, SD = 90.11) than incongruent trials (*M* = 547.26 ms, SD = 89.19) (Figure 4B).

#### Accuracy

**Figure 5:**
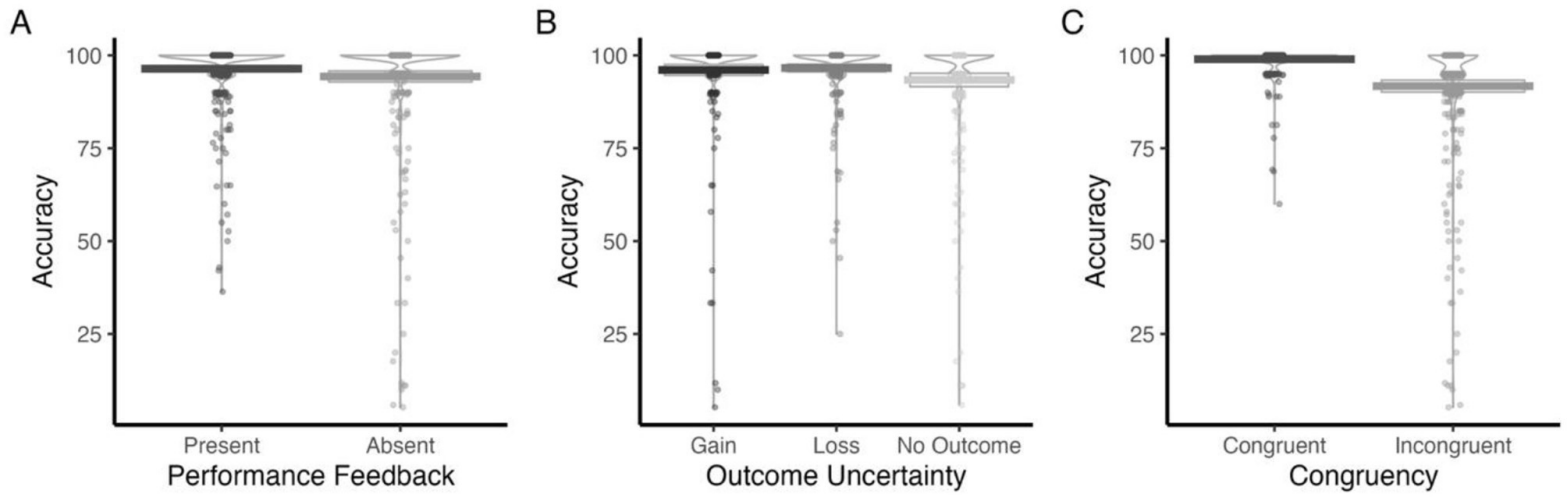
Mean Accuracy, split by: Performance Feedback **(A)**, Outcome Uncertainty **(B)**, and Congruency **(C)**.

As depicted in Table 4, accuracy varied as a function of Performance Feedback. As such, participants exhibited a greater overall accuracy when feedback was provided (*M* = 96.39, SD = 8.72), compared to when it was withheld (*M* = 94.32, SD = 15.36) (Figure 5A). Accuracy further differed by Outcome Uncertainty. More specifically, participants were significantly more accurate in both Loss (*M* = 96.6, SE = 0.86, *p* = 0.0014) and Gain conditions (*M* = 96.1, SE = 0.86, *p* = 0.01), relative to the No Outcome condition (*M* = 93.4, SE = 0.86) (Figure 5B). No significant difference was found between Loss and Gain (*p* = 0.826). Furthermore, congruency exerted a strong influence on performance, with higher accuracy on congruent trials (*M* = 98.98, SD = 3.79) than incongruent trials (*M* = 91.73, SD = 16.53) (Figure 5C).

**Figure 6:**
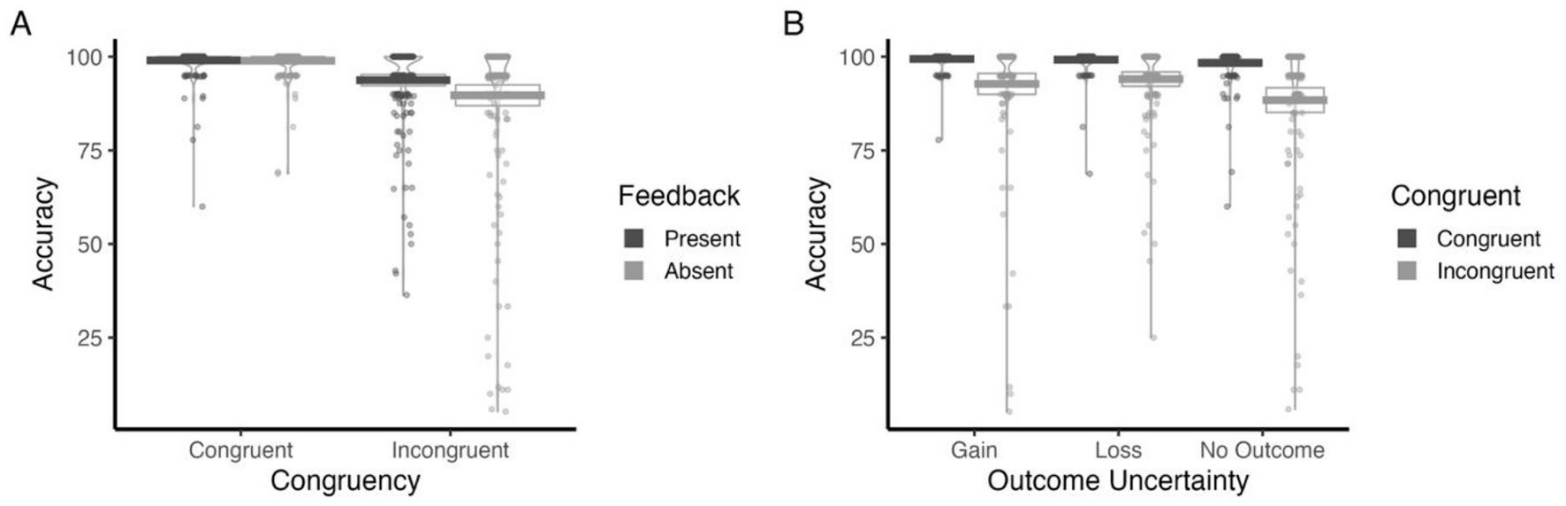
Congruent and Incongruent Accuracy for feedback-present and feedback-absent trials **(A)**, alongside mean Accuracy for Gain, Loss and No Outcome conditions **(B)**, separated by Congruency (Congruent, Incongruent).

*Post-hoc* analyses performed to explore the significant Performance Feedback × Congruency interaction (Table 4) revealed that in congruent trials, the presence of feedback had no significant impact on accuracy (*b* = 0.10, SE = 1.06, *t*_(818)_ = 0.09, *p* = 0.925). However, when faced with incongruent trials, feedback significantly improved task accuracy (*b* = 4.06, SE = 1.06, *t*_(818)_ = 3.82, *p* = 0.0001) (Figure 6A). In turn, during feedback trials, accuracy was significantly higher on congruent than incongruent trials (*b* = 5.27, SE = 1.06, *t*_(818)_ = 4.96, *p* < 0.0001). This congruency effect was even larger in the absence of feedback (*b* = 9.23, SE = 1.06, *t*_(818)_ = 8.68, *p* < 0.0001) (Figure 6A).

Outcome Uncertainty also interacted with Congruency (Table 4). Follow-up comparisons revealed that for congruent trials, there were no significant differences between outcome types (all ps > 0.70). However, for incongruent trials, significant differences emerged: participants performed significantly better on Loss trials compared to No Outcome trials (*M* = 5.61, SE = 1.30, *t*_(818)_ = 4.31, *p* < 0.001), and significantly better on No Outcome trials compared to Gain trials (*M* = −4.33, SE = 1.30, *t*_(818)_ = −3.33, *p* = 0.003) (Figure 6B). The difference between Loss and Gain trials was not significant (*p* = 0.589). Further analysis showed that the congruency effect was significant across all outcome types: participants performed better on congruent than incongruent trials for Loss outcomes (*M* = 5.17, SE = 1.30, *t*_(818)_ = 3.97, *p* < 0.001), No Outcome trials (*M* = 9.94, SE = 1.30, *t*_(818)_ = 7.63, *p* < 0.001), and Gain trials (*M* = 6.65, SE = 1.30, *t*_(818)_ = 5.10, *p* < 0.001), with the largest effect observed for No Outcome trials (Figure 6B).

### Individual differences based on intolerance of uncertainty

#### Valence

A significant interaction emerged between Outcome Uncertainty and Intolerance of Uncertainty (IUS) scores (Table 2). To clarify this effect, a simple slopes analysis was conducted to examine how the relationship between IUS and valence ratings varied across Outcome Uncertainty conditions. Pairwise comparisons of estimated slopes indicated a significant difference between the Gain and Loss conditions: higher IUS scores were more strongly associated with reduced valence ratings in the Gain context relative to the Loss context (*b* = 0.049, SE = 0.019, *t*_(372)_ = 2.55, *p* = 0.030). In contrast, differences between the Loss and No Outcome conditions (*b* = 0.035, SE = 0.019, *t*_(372)_ = 1.90, *p* = 0.141), as well as between the No Outcome and Gain conditions (*b* = 0.012, SE = 0.019, *t*_(372)_ = 0.65, *p* = 0.792), were not statistically significant.

Finally, a significant three-way interaction was found between Performance Feedback, Outcome Uncertainty and IUS. Follow-up analyses revealed that IUS slope was significantly steeper when feedback was provided in Loss contexts, compared to feedback-absent Gain contexts, *b* = 0.08, SE = 0.03, *t*_(372)_ = 3.05, *p* = 0.029. This indicates that individuals higher in IUS reported more negative valence ratings when encountering Loss blocks, accompanied by performance feedback.

#### Response time

Individual differences in IUS significantly predicted response speed (Table 4), with higher IUS scores associated with faster reaction times across the task (β = −3.21, SE = 1.34, *t*_(91.11)_ = −2.39, *p* = 0.019).

A significant two-way interaction between Performance Feedback and IUS was identified (Table 4). Follow-up analyses indicated that when feedback was presented, higher IUS scores were associated with slower reaction times compared to when feedback was absent (*b* = −0.81, *SE* = 0.31, *t*_(818)_ = −2.65, *p* = 0.008).

#### Accuracy

Results from further examination of the significant Congruency × IUS interaction (Table 4) revealed that IUS exerted a significantly stronger negative effect on accuracy under incongruent, relative to congruent conditions (β = 0.225, SE = 0.099, *t*_(818)_ = 2.25, *p* < 0.025).

### Individual differences based on anxiety and depression symptoms

#### Valence ratings

Among the individual difference measures, Anhedonic Depression, as measured by the MASQ-AD, exhibited a significant main effect (Table 2). As such, higher scores were associated with more negative valence ratings, β = −0.12, SE = 0.04, *t*_(154.91)_ =-2.72, *p* = 0.007.

#### Flanker fixation

Anxious Arousal, as indexed by the MASQ-AA, significantly predicted fixation patterns (Table 3): greater levels of Anxious Arousal were associated with increased flanker fixations across the task, β = 3.15, SE = 0.98, *t*_(79.4)_ = 3.2, *p* = 0.002.

Although analyses reveal a significant interaction between Outcome Uncertainty and MASQ-AA, MASQ-GD and MASQ-AD (Table 3), these effects did not remain when entering these individual difference measure separately. Therefore, these effects were not followed up.

Furthermore, both Performance Feedback and Outcome Uncertainty significantly interacted with MASQ-AA and MASQ-AD scores (see Table 3). For MASQ-AA, follow-up comparisons of simple slopes revealed that, in the absence of feedback, MASQ-AA slope was significantly more negative in the Gain condition compared to both the Loss (Δ = −1.469, SE = 0.444, *t*_(664)_ = −3.309, *p* = 0.013) and No Outcome conditions (Δ = −1.4885, SE = 0.444, *t*_(664)_ = −3.352, *p* = 0.011). This pattern suggests that participants reporting higher levels of Anxious Arousal showed reduced attention to flankers in Gain contexts, but only when Performance Feedback was not provided. No other slope comparisons reached statistical significance (all ps > 0.07). For MASQ-AD, when feedback was provided, individuals with higher Anhedonic Depression scores showed a significantly stronger positive association with flanker fixations in the Loss condition compared to the No Outcome condition (Δ = 1.1161, SE = 0.368, *t*_(664)_ = 3.029, *p* = 0.03). Additionally, the association between MASQ-AD and flanker fixations was significantly more positive in the Gain condition relative to the No Outcome condition (Δ = −1.2243, SE = 0.368, *t*_(664)_ = −3.323, *p* = 0.012). These effects were only observed during feedback trials. No other comparisons reached statistical significance (ps > 0.1).

#### Feedback fixation

The linear mixed model revealed a significant main effect of MASQ-AA (Table 3), further examination of this result showed that across all conditions, higher MASQ-AA scores were significantly associated with more fixations (*b* = −0.1037, SE = 0.049, *t*_(154.914)_ = −2.099, *p* = 0.04).

While analyses showed a significant interaction between Outcome Uncertainty and MASQ-AD and MASQ-GD (Table 3), these effects did not persist when these individual difference measures were analyzed separately. As such, no further investigation of these effects was undertaken.

A significant three-way interaction between Outcome Uncertainty, Performance Feedback, and MASQ-AA was found (Table 3). Specifically, in the feedback-absent Gain condition, compared to the feedback-absent No Outcome condition, greater Anxious Arousal was significantly associated with fewer fixations on the feedback cue (*b* = −1.29, SE = 0.396, *t*_(666)_ = −3.26, *p* = 0.015). No other pairwise comparisons reached statistical significance, including those within the feedback-present conditions (all ps > 0.10).

#### Response time

Another of the individual difference measures, MASQ-AA, showed a significant interaction with trial Congruency (Table 4). Specifically, higher levels of Anxious Arousal were associated with faster responses on congruent trials relative to incongruent trials (Δ = −1.29, SE = 0.55, *t*_(818)_ = −2.41, *p* = 0.018).

Although MASQ-AD and MASQ-GD initially showed significant interactions with Performance Feedback and Outcome Uncertainty (Table 4), these effects did not withstand correction in *post-hoc* analyses and should be interpreted cautiously.

#### Accuracy

Accuracy also varied as a function of Congruency and MASQ-AD scores (Table 4). As such, MASQ-AD scores were more positively associated with accuracy in the congruent condition than in the incongruent condition (*b* = 0.312, SE = 0.161, *t*_(818)_ = 1.94, *p* = 0.053).

## Discussion

The current study examined how outcome uncertainty related to gain and loss, information availability via performance feedback, and individual differences impacted motivational and emotional phenomena during a cognitive control task i.e., modified Eriksen Flanker. Task blocks with potential gain and loss, relative to no outcome, resulted in higher ratings of arousal and higher task accuracy. In addition, tor task blocks with potential loss, relative to no outcome, reaction times were faster. Task blocks with the absence, relative to presence of performance feedback, led to lower ratings of arousal, lower ratings of positive valence, lower task accuracy, and fewer fixations during the flanker presentation There was little evidence for interactions between outcome uncertainty related to gain and loss, and performance feedback during the cognitive control task. Individual differences in intolerance of uncertainty, anxious arousal, and anhedonic depression were related to multiple read-out measures during the task. Overall, these findings suggest that outcome uncertainty, performance feedback, intolerance of uncertainty, and anxious/depressive traits have different effects upon motivational and emotional phenomena.

### General effects of outcome uncertainty related to gain and loss

There was sufficient evidence for H1—that outcome uncertainty related to gain and loss, relative to no outcome, would lead to higher affect and motivation (Grupe and Nitschke, 2013; Morriss et al., 2019). Task blocks with potential gain and loss, relative to no outcome, resulted in higher ratings of arousal and higher task accuracy (Hübner and Schlösser, 2010). For task blocks with potential loss, relative to no outcome, reaction times were also faster. Lastly, no differences were observed based on the valence of the outcome for fixation count. In sum, these findings indicate that outcome uncertainty related to gain and loss generally evokes the relevant and expected emotional experience and motivational system (e.g., aversive and appetitive) during cognitive control. Such findings are in line with previous research demonstrating uncertain reward and punishment stimuli during passive viewing to evoke a range of emotions and motivational engagement, indexed by different read-outs (for reviews see Grupe and Nitschke, 2013; Morriss et al., 2019).

### General effects of performance feedback

There was evidence for H2—that the lack of possibility to reduce uncertainty related to the absence, relative to the presence of performance feedback would lead to lower positive affect and motivation. For instance, task blocks with the absence, relative to presence of performance feedback, led to lower ratings of arousal, lower ratings of positive valence, lower task accuracy, and fewer fixations during the flanker presentation. Furthermore, the absence, relative to the presence of performance feedback during incongruent trials resulted in poorer task accuracy, similar to previous research using cognitive control tasks (Moret-Tatay et al., 2016). However, the absence or presence of performance feedback did not appear to modulate reaction times or fixation count during the presentation of feedback. The findings here suggest that the absence of performance feedback may lower the chance for reducing uncertainty, which may help explain the observed dampening of arousal, positive valence and motivation (Morriss et al., 2022), particularly when task demand was high, within these experimental blocks. Such findings sit alongside previous literature that has demonstrated that under uncertainty there is greater desire for receiving performance feedback (Anseel and Lievens, 2007).

### Interactions between outcome uncertainty related to gain and loss and performance feedback

Interestingly, there was little evidence for H3—that there would be interactions between outcome uncertainty and performance feedback on the different read-out measures. The findings reported in this study are at odds with the existing literature on uncertain threat and reward stimuli during passive viewing, where there are clear interaction effects on different read-outs (Foti and Hajcak, 2012; Lin et al., 2015; Morriss et al., 2019). The differences in findings may reflect the differences in context, given that the cognitive control task was relatively easy (e.g., accuracy was generally high for the congruent condition) and involved low stakes (e.g., small monetary incentive). Perhaps, the combined impact of outcome uncertainty and performance feedback in the cognitive control task would be more apparent if the task difficulty and the stakes were higher. To summarize, from these findings there is not enough evidence either way to suggest that outcome uncertainty related to gain and loss and performance feedback under task demand elicits greater emotional experience or that both the aversive and appetitive systems are engaged equally.

### Individual differences based on intolerance of uncertainty

Notably, the findings varied for H4, which was based on individual differences in intolerance of uncertainty. Intolerance of uncertainty modulated responses based on the absence or presence of performance feedback. More specifically, higher intolerance of uncertainty was associated with faster reaction times to flankers in blocks where there was an absence vs. presence of feedback. This finding is in line with past research suggesting that those with higher intolerance of uncertainty are more motivated to resolve uncertainty quickly (Fergus and Carleton, 2016). In this study, intolerance of uncertainty also modulated responses on the basis of the outcome uncertainty for gain and loss outcomes during the cognitive control task, where lower intolerance of uncertainty was associated with greater valence rating differentiation between loss vs. gain, and loss vs. no outcome task blocks. Such that individuals with lower, relative to higher intolerance of uncertainty rated task blocks with potential loss outcomes more negatively, and task blocks with potential gain outcomes more positively. These findings suggest that individuals with higher intolerance of uncertainty tend to generalize their experiences of valence under outcome uncertainty, regardless of whether the outcome was based on gain or loss, which is in line with the intolerance of uncertainty and threat generalization literature (Bauer et al., 2020; Morriss et al., 2016). Interestingly, higher intolerance of uncertainty was also associated with more negative valence ratings during loss blocks with feedback, suggesting that negative affect was more clearly experienced when there was feedback about loss outcomes. Lastly, higher intolerance of uncertainty was also associated with faster correct reaction times overall during the cognitive control task. As far as we are aware there are no other findings like this one in the literature. Such a finding may reflect that intolerance of uncertainty is related to greater attentional vigilance in general, which has been demonstrated in a few studies with different read-outs (Fergus and Carleton, 2016; see supplemental materials for Huang et al., 2023). However, this finding needs to be interpreted with caution because intolerance of uncertainty was not found to modulate fixation count in this study, which is another index of attentional vigilance. Thus, further replication is required. Taken together, these findings indicate that to some extent intolerance of uncertainty exacerbates emotional and motivational phenomena, namely via self-report and behavior, when the environment includes task demand with uncertainty and mixed valence outcomes.

### Individual differences based on anxiety and depression symptoms

With regards to H5, which was based on symptoms of anxious arousal and anhedonic depression, there were a few standout findings. Greater symptoms of anxious arousal were associated with a higher fixation count to the flanker targets and feedback cues overall, in line with the account that attentional vigilance is heightened during anxious states (Mogg and Bradley, 1998). Interestingly, higher anxious arousal was also associated with fewer fixations to the flanker and feedback cues during the no feedback gain block suggesting that anxious arousal lowers the seeking of uncertain reward. Conversely, higher anhedonic depression was associated with more fixations to the flanker during gain and loss blocks with feedback, suggesting that those experiencing anhedonia are more motivated by potential gain and loss outcomes when there is greater certainty of them happening. Lastly, greater symptoms of anhedonic depression was associated with more negative valence ratings across task blocks regardless of the outcome, which supports the account that positive affect is dampened during anhedonic states (Treadway and Zald, 2011). However, symptoms of anxious arousal and anhedonic depression were not observed to interact with any other read-outs. In sum, these findings suggest that current symptoms of anxiety and depression are involved in altering emotional and motivational phenomena, specifically self-report and fixation count, when the environment includes task demand with uncertainty and mixed valence outcomes.

### Implications, strengths, limitations, and conclusions

While this study provides insight into how emotional and motivational phenomena are impacted by these different factors, questions remain on the generalizability of results. For instance, are these findings specific or would similar findings emerge under different forms of attention (e.g., alerting, orienting) or other cognitive processes (e.g., working memory). There are opportunities for future studies, potentially multi-lab efforts, to examine such questions by using the same experimental setu*p* with small modifications (e.g., change the parameters of uncertainty, gain and loss, task difficulty). Addressing these questions would better our understanding of how emotional and motivational phenomena are impacted by outcome uncertainty related to gain and loss, performance feedback, and individual differences, as well as have implications that extend beyond basic science (e.g., occupational psychology, educational psychology, clinical practice).

The study reported here had a few notable strengths. Firstly, the study used a well-established cognitive control task (Erikson Flanker), multiple read-out measures, and several individual difference measures. Secondly, the study recorded ratings both on the discrete emotional states and dimensions of valence/arousal, providing further detailed insight into how these aspects of emotion are related to outcome uncertainty and performance feedback. Thirdly, even though the study was conducted online, we were able to interact with the participants to check that they understood what to do during the experiment. The study also had a few limitations with regards to using eye-tracking for online experiments. As previously described, fixation count, irrespective of region-of-interest, did not differ as a function of outcome uncertainty or performance feedback. This finding may echo potential problems with the quality of the eye-tracking data due to a lower sampling rate and lack of recalibration. For instance, although a researcher was initially present to assess the testing environment and reinforce eye-tracking requirements (e.g., minimize head movement, calibration)—these may not have been maintained throughout the course of the task. This exemplifies a key consideration when choosing to conduct online vs. in-per*s*on research: despite online research bearing many advantages (e.g., speed, convenience and affordability), there is an inherent lack of control. For this finding, this may have been rectified, in part, by having recalibration and validation phases between blocks (Yang and Krajbich, 2021). Another limitation was that response time dependencies and post-error slowing were not included as covariates for RT analyses (Viviani et al., 2024).

In conclusion, the general pattern of results from this study suggests that during a cognitive control task: (1) uncertainty about task performance through the absence of performance feedback dampened arousal and positive affect, and led to demotivation (i.e., lower fixation count during the presentation of flankers and poorer task accuracy, (2) outcome uncertainty related to loss and gain evoked greater arousal and engaged motivation (i.e., higher task accuracy), (3) higher intolerance of uncertainty is associated with less discrimination between valence ratings for outcome uncertainty related to gain and loss, and higher motivational engagement (i.e., faster reaction time) during the task generally and particularly when feedback is absent, and (4) anxious symptoms are associated with increased motivation across the task (e.g., greater fixation count), and (6) anhedonia symptoms are associated with general negative affect throughout the task. Future research on how outcome uncertainty, performance feedback, and individual differences impact emotional and motivational phenomena would benefit from replicating or modifying the experiment used in this study to assess the generalizability of these results to other attentional or cognitive processes.

## Data Availability

Publicly available datasets were analyzed in this study. This data can be found here: The merged data file and analyses are available here: https://osf.io/hm6r9/.
